# Meeting of Dentists at Louisville

**Published:** 1883-04

**Authors:** 


### EDITORIAL.

### Meeting of Dentists at Louisville,

At a meeting of the Dentists of the city of Louisville, held at the Dental Depot of Dr. Frazee, on Monday, Mar. 5th, Dr. W. G. Redman was called to the chair, and Dr. C. E. Dunn made Secretary.

The Chairman announced that the object of the meeting was, to take some action in memory of Dr. W. H. Goddard, who died at his home in this city on Sunday, Mar. 4th, at one and three quarter hours, A. M.

On motion, Drs. C. E. Dunn, F. Peabody and B. O. Doyle, were appointed a Committee to draft resolutions.

The Committee on resolutions submitted their report, which was on motion received and adopted, viz:

God having called from among us, our friend and brother, Dr. W. H. Goddard; while we with humble reverence submit to the will of Him, who doeth all things well, it is fitting that we assemble together to do honor to one, who had for so many years, honored our profession.

Dr. Goddard entered the Dental profession in its infancy, and labored earnestly and faithfully to advance and bring it to that high standard, to which he felt it was worthy and justly entitled, and to the day of his death held its interests near to heart, and constantly in mind.

He opened an office in our city, about the year 1840, and, with the exception of a short time that he was engaged in mercantile pursuits, was in active practice. A few years since, the weight of years caused him to retire, but not to lose his love and interest in the profession with which he had so long been connected. A few weeks before his death, he expressed his fears that he would not get to Niagara, to the meeting of the "American Dental Association" (showing how constantly it was in his thoughts) in which Association he had for fifteen successive years, been elected its Treasurer, performing the duties of that trying position so faithfully, that he was familiarly called, "The Watch Dog of the Treasury." In August last, at its meeting in Cincinnati, he was elected President, holding that position at his death. We can truly say of him, he died with the harness on.

In his death, the members of our profession have lost a bright example, a good counselor and true friend, to whom we could always apply freely for advice and help, and while it might sometimes be given in his short blunt way, we well knew it was honest and could be depended on.

In token of our love and respect for him, we inscribe these few lines to his memory, and order that they be published in the Dental Journals, and a copy furnished his family, and that we attend the funeral in a body.

CHAS. E. DUNN.

F. PEABODY. } COMMITTEE.

B. OSCAR DOYLE.

Attest:

CHAS. E. DUNN, Secy. W. G. REDMAN. Chairman

